# Case Report: Primary Leptomeningeal Medulloblastoma in a Child: Clinical Case Report and Literature Review

**DOI:** 10.3389/fped.2022.925340

**Published:** 2022-07-11

**Authors:** Daria Morgacheva, Alexandra Daks, Anna Smirnova, Aleksandr Kim, Daria Ryzhkova, Lubov Mitrofanova, Alena Staliarova, Evgeniya Omelina, Alexey Pindyurin, Olga Fedorova, Oleg Shuvalov, Alexey Petukhov, Yulia Dinikina

**Affiliations:** ^1^Almazov National Medical Research Centre, Saint Petersburg, Russia; ^2^Laboratory of Gene Expression Regulation, Institute of Cytology, Russian Academy of Sciences, Saint Petersburg, Russia; ^3^Hematology and Immunology, Oncological Department 3, Belarusian Research Center for Pediatric Oncology, Minsk, Belarus; ^4^Laboratory of Cell Division, Department of Regulation of Genetic Processes, Institute of Molecular and Cellular Biology, Siberian Branch of Russian Academy of Sciences, Novosibirsk, Russia

**Keywords:** primary leptomeningeal medulloblastoma, pediatric cancer, next-generation sequencing, central nervous system tumors, oncology

## Abstract

Medulloblastoma is one of the most common pediatric central nervous system malignancies worldwide, and it is characterized by frequent leptomeningeal metastasizing. We report a rare case of primary leptomeningeal medulloblastoma of an 11-year-old Caucasian girl with a long-term disease history, non-specific clinical course, and challenges in the diagnosis verification. To date, 4 cases of pediatric primary leptomeningeal medulloblastoma are reported, and all of them are associated with unfavorable outcomes. The approaches of neuroimaging and diagnosis verification are analyzed in the article to provide opportunities for effective diagnosis of this disease in clinical practice. The reported clinical case of the primary leptomeningeal medulloblastoma is characterized by MR images with non-specific changes in the brain and spinal cord and by 18FDG-PET/CT images with diffuse heterogeneous hyperfixation of the radiopharmaceutical along the whole spinal cord. The immunohistochemistry and next-generation sequencing analyses of tumor samples were performed for comprehensive characterization of the reported clinical case.

## Introduction

Medulloblastoma (MB) is the most common primary tumor of the central nervous system (CNS) in children and accounts for about 20% of all neoplasms of the CNS and 63% of intracranial embryonal tumors (1–3). In 70% of cases, MB occurs in children under 10 years of age, while the age-specific incidence has two peaks at the age of 1–4 years and 5–9 years with male predominance (3).

Primary MB is almost exclusively located in the hemispheres or in the vermis of the cerebellum and is characterized by highly invasive and aggressive growth (2). One of the specific characteristics of MB is a tendency of metastasizing through cerebrospinal fluid (CSF) pathways, and at the time of initial diagnosis, about 35% of patients have a metastatic stage (3). MB is always classified as a grade IV tumor due to its high malignant potential and rapid growth rate (4).

Primary leptomeningeal MB is an extremely rare form and is characterized by diffuse leptomeningeal lesions in the absence of primary masses in the cerebellum. The complexity of diagnosis of primary leptomeningeal form is explained by atypical clinical manifestations and difficulties of differential diagnosis. To the best of our knowledge, this is the fifth reported case of the primary pediatric leptomeningeal form of MB worldwide (Table 1). All of the previously reported cases were also associated with unfavorable outcomes.

**TABLE 1:** Reported cases of pediatric primary leptomeningeal MB.

Year of report	Age, sex	Symptoms	Initial radiological picture	Surgical intervention, chemotherapy	Histological and molecular subgroup	Outcome	References
1989	5, M	Headache, vomiting, decreased consciousness	Widespread diffusion in the subarachnoid spaces of the posterior fossa	Craniectomy	MB	Fatal	(15)
2007	10, F	Increasing headaches, vomiting, altered sensorium	Lesions of all posterior fossa leptomeninges	Lateral suboccipital craniectomy and biopsy; adjuvant chemotherapy	MB	Expired after 2 weeks after surgery	(16)
2009	8, M	Headache, progressive amblyopia during 2 months	Lesion of the meninges in the projection of the cerebellar folia and convexity surfaces of the brain without intraparenchymal lesions	Suboccipital craniotomy, biopsy; 2 chemotherapy cycles (the Head Start III protocol)	MB	Death 8 months after the onset of the disease	(17)
2018	11, M	Occipital dominant headaches for a year. Generalized tonic spasm and upward tonic deviation of eyes. An episode of projectile vomiting.	Massive hydrocephalus. Nodular pachymeningeal enhancement in cerebellar hemisphere	Emergency surgical decompression, suboccipital craniectomy, biopsy. Radiation and chemotherapy	MB, classic non-WNT/non-SHH	The patient was stable and received the therapy at the time of publication	(18)

*The table includes all reported cases of pediatric primary leptomeningeal form of MB. M, male; F, female; MB, medulloblastoma.*

To date, it is generally accepted that MB is a heterogeneous group of CNS tumors with different phenotypes, molecular characteristics, responses to anticancer therapy, and clinical outcomes. It is important to mention that for subgrouping MB, both histological and molecular genetic approaches are used to define treatment strategy and prognosis (4–6).

Due to the primary location of MB in the posterior fossa, symptoms and complaints caused by MB are mostly non-specific and may indicate a wide range of diseases that delay the diagnosis and initiation of the specific treatment. In 20–40% of cases, the predominant clinical symptom of MB is the progression of hypertensive-hydrocephalic syndrome that manifests with headaches, nausea, vomiting, seizures, and depression of consciousness (7, 8). In addition, MB frequently causes the disorders of cerebrospinal fluid (CSF) circulation, which requires immediate treatment, including drainage by surgical implantation of the CSF shunt system (9). Other manifestations, depending on the localization of the primary tumor and metastases, are uncoordinated movements, cranial nerves palsy, nystagmus, diplopia, and restricted eye movements (10, 11).

The typical radiological signs of MB are the presence of a clearly defined mass of the posterior cranial fossa or cerebellum, corresponding to the primary localization, often rounded, with calcifications, cysts and areas of necrosis, and peritumoral edema, frequently with heterogeneous contrast enhancement. Common forms of MB chiefly show isointensity on T2-WI and fluid-attenuated inversion recovery (FLAIR), high intensity on D-WI, and low intensity on apparent diffusion coefficient (ADC) MR images and also demonstrate strong signal intensity on T1-CE after contrast injecting (12). Leptomeningeal foci usually indicate a metastatic form of MB; but in rare cases, it could be the primary lesions. It has been shown that the specific combinations of MRI signs correspond to concrete histological types (13) and even molecular subgroups of MB (14).

Today, the standard treatment of MB is a combination of surgical resection, chemotherapy, and radiation therapy. The therapeutic strategies are various and depend on the risk group of the disease, the age of the patient at the time of diagnosis, as well as the extent of surgical resection and response to chemotherapy. At the same time, there should be noted a tendency toward reducing the intensity of the anticancer treatment in groups with a favorable prognosis and in young children in order to minimize acute and long-term toxicity.

In this article, we report the first case of pediatric primary leptomeningeal medulloblastoma in Russia. The approaches of neuroimaging, histological, and molecular methods of diagnosis were applied to improve the diagnosis of rare cases in clinical practice.

## Case Description

An 11-year-old Caucasian girl with severe neurological symptoms was hospitalized at the Department of Pediatric Neurosurgery, Almazov National Medical Research Centre, St Petersburg, in June 2020. By the time of hospitalization, the patient’s medical history was 5 months. In March 2020, the patient was admitted to the local hospital with increased fatigue, febrile fever, headaches accompanied by periodic vomiting, diplopia, and decreased visual acuity.

With suspected meningoencephalitis, the patient was admitted for inpatient antibacterial treatment during which the progression of cerebral symptoms up to sopor has been observed. According to the brain and spinal cord MRI with contrast enhancement, no data for the tumor mass were obtained. Occlusive triventricular hydrocephalus, periventricular edema, and diffuse structural changes in the meninges with the signs of inflammation that are typical to leptomeningitis were diagnosed. CSF cytology did not reveal any pathology. To relieve progressive hypertensive-hydrocephalic syndrome, therapy with systemic corticosteroids was initiated, and ventriculoperitoneal shunting was performed. After the surgery was provided, reduction of neurological symptoms and clinical improvement of patient’s condition were observed.

**FIGURE 1:**
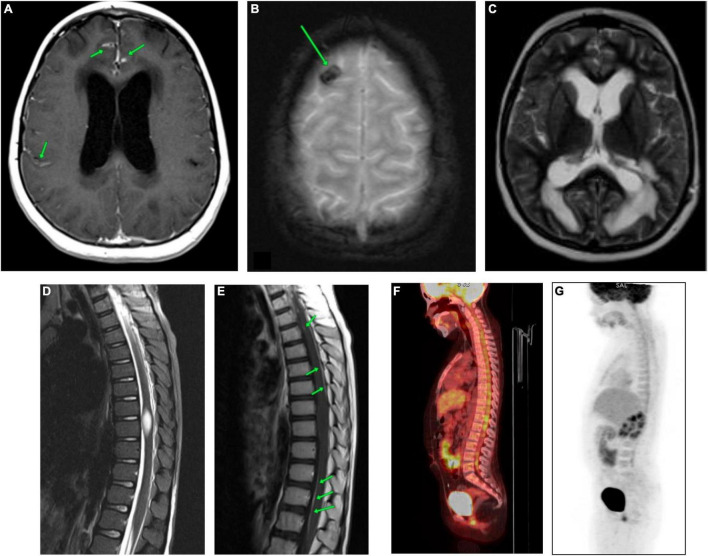
CNS MRI and the whole-body PET-CT of the patient carried out at the time of admission to the Almazov National Medical Research Centre. **(A)** Brain MRI (CE T1-WI). Diffuse infiltration of the meninges (soft and hard) of the cerebral hemispheres with uneven thickening and contrast accumulation. **(B)** Brain MRI (T2*-WI). The area of hemosiderin deposition in the cortex of the right frontal lobe. **(C)** Brain MRI (T2-WI). Triventricular hydrocephalus. Ventriculoperitoneal shunting (VPS), the shunt in the left lateral ventricle. Severe periventricular edema. Subarachnoid spaces are narrowed. **(D)** Spinal cord MRI (STIR). The spinal cord at the level of Th8-Th9 vertebrae is thickened, and a spindle-shaped formation of 22 mm x 9 mm x 8 mm is observed. **(E)** Spinal cord MRI (post-contrast T1-WI). Pia mater of the thoracic region of spinal cord evenly accumulates a contrast agent. The spindle-shaped formation shows no signs of contrast. **(F,G)** The whole-body [18F]2-fluoro-2-deoxy-D-glucose positron emission tomography/computed tomography (18FDG-PET-CT). The picture of diffuse heterogeneous hyperfixation of the radiopharmaceutical along the course of the cervical, thoracic, and lumbar segments of the spinal cord with a maximum value of standardized drug capture of 3.12.

At the time of hospitalization at the Almazov National Medical Research Centre, the patient had moderate asthenic syndrome and ataxia. MRI of the brain and spinal cord showed diffuse infiltration of the pia mater and dura mater of the cerebral hemispheres and posterior cranial fossa (PCF) with irregular thickening and contrast enhancement (Figure 1A and Supplementary Figures 1A,B).

Regional edema, cystic transformation, and hemorrhagic impregnation of cerebellar hemispheres, as well as a site of hemosiderin deposition in the cortex of the right frontal lobe, were visualized (Figure 1B and Supplementary Figures 2A,B). Symmetrical expansion of the lateral ventricles and III ventricle, as well as severe periventricular edema, was detected, while the subarachnoid spaces were narrowed (Figure 1C and Supplementary Figure 3).

In the spinal cord, a spindle-shaped lesion with the size of 20 mm × 9 mm × 8 mm (Figure 1D and Supplementary Figure 4) at the level of Th8-Th9 vertebrae was revealed, with hyperintense MR signal on T2-WI and STIR and without contrast enhancement (Figure 1E), which has been regarded as myelitis. The contrast enhancement by the pia mater was noted throughout the thoracic spinal cord (Figure 1E). Repeated CSF cytology was normal.

Considering non-specific MRI data, poor accessibility for biopsy, and high risks of neurological complications, as well as stable patient’s condition due to the conservative therapy [acetazolamide 250 mg/day, prednisolone 1 mg/kg/day (35 mg)], it was decided to continue the diagnostic search.

The whole-body [18F]2-fluoro-2-deoxy-D-glucose positron emission tomography/computed tomography (18FDG-PET/CT) allowed to reveal diffuse heterogeneous hyperfixation of the radiopharmaceutical along the cervical, thoracic, and lumbar spinal cord segments with a maximum standardized uptake of 3.12. This PET-CT picture was non-specific and could be observed in both tumor lesions and inflammatory processes (Figures 1F,G).

The patient was continuously treated with combined therapy that included decongestant, desensitizing, antibacterial, immunomodulation, neurometabolic, and steroid medications, and her condition remained stable for 1 month.

However, upon the attempt to reduce the doses of steroid therapy, there was a deterioration of neurological symptoms with a progressive disturbance of consciousness level, increased ataxia, stiffness of the occipital muscles, paraparesis of the lower extremities to 2 points, accession of ptosis of the right eyelid, and decrease in visual acuity.

**FIGURE 2:**
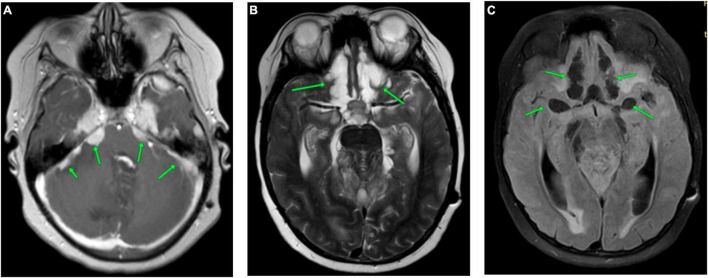
CNS MRI of the patient after 5.5 months of the debut of the disease. **(A)** Brain MRI (post-contrast CE T1-WI). Accumulation of contrast agent by thickened meninges in cerebellar tentorium (green arrows). **(B)** Brain MRI (T2-WI). **(C)** Brain MRI (TIRM). Significant increase in cystic lesions of the frontal lobes (green arrows).

The subsequent MRI examinations revealed an increased size of nodular thickenings of dura mater in the area of the tentorium cerebelli (Figure 2A and Supplementary Figures 5A,B); increased cerebral edema in the area of frontal lobes, in the poles of the temporal lobes and cerebellar hemispheres; persisted and progressed diffuse infiltrative changes in the meninges; and increased zones of cystic transformation of the medulla in the cerebellum and frontal lobes (Figures 2B,C and Supplementary Figure 6). Due to the impairment of the patient’s status and negative dynamics of neuroimaging data, a biopsy of the pathological region of the cerebellar pia mater was performed (6 months after the clinical manifestation of the disease).

**FIGURE 3:**
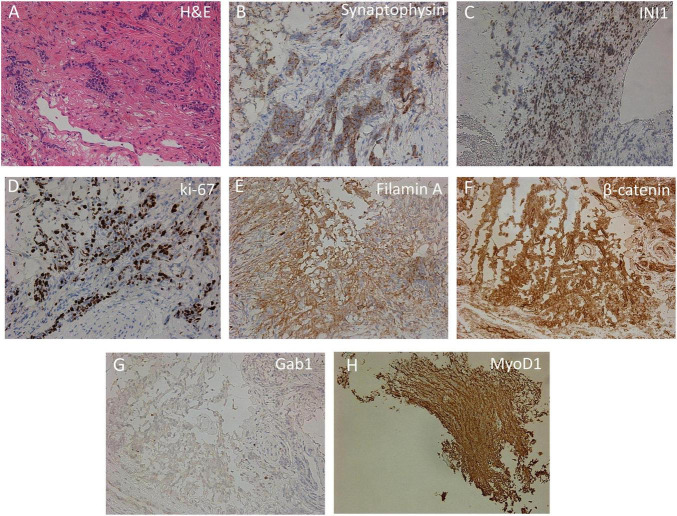
Immunohistochemical analysis of cortical biopsy. **(A)** Hematoxylin-eosin staining (H,E). **(B–H)** Immunohistochemistry using anti-synaptophysin **(B)**, anti-INI1 **(C)**, anti-Ki-67 **(D)**, anti-filamin A **(E)**, anti-β-catenin **(F)**, anti-Gab1 **(G)**, and anti-MyoD1 **(H)** antibodies.

As a result of the histomorphological examination, among the fibrous tissue, the complexes of small atypical cells with a narrow rim of the cytoplasm and hyperchromic nuclei were identified (Figure 3A). Tumor cells showed positive staining with antibodies against synaptophysin, MyoD1, filamin A, β-catenin, INI1, and GAB1 (Figure 3). Ki-67 staining revealed 70% positive cells. Based on the obtained histomorphological and immunohistochemical data, leptomeningeal medulloblastoma Grade IV, WNT molecular subgroup was diagnosed. Adjuvant therapy was not carried out due to the progressive impairment of the patient’s condition, and the patient died 42 days after the biopsy.

## Discussion

The primary leptomeningeal form of MB is an extremely rare malignancy and is associated with tumor aggressiveness and an unfavorable prognosis. The non-specific clinical picture, combined with the difficulties of diagnosis verification, led to a long diagnostic period, which also causes significant deterioration of the prognosis. In the analyzed clinical case, there was a 6-month interval from the manifestation of the disease to the hospitalization in a specialized oncology department where the diagnosis has been verified.

According to the literature, this is the fifth reported clinical case of the primary leptomeningeal form of MB in a child (Table 1). At the moment of clinical manifestation of the disease, almost all patients had cerebral symptoms (15–18). The median age at diagnosis was 9 years (range 5–11 years) with male predominance (15–18). MRI scans showed predominantly meningeal lesions with contrast enhancement but there were no definite lesions in the brain parenchyma (15–18). All patients underwent a biopsy with a subsequent histological examination that verified the diagnosis of MB (15–18). Given the years of published cases, the identification of the molecular subgroup of MB was performed only in one case, and the subgroup non-WNT/non-SHH was defined (18). All patients died a few weeks after the manifestation of the disease.

We report this case to pay attention to the features of the manifestation and hence to increase the chances of its timely diagnosis in the future. The complexity of clinical and laboratory data makes it possible to suspect the oncological diagnosis. Attention should be paid to the presence of cerebral symptoms at the onset of the disease (headaches accompanied by periodic vomiting, double vision, and deterioration of visual acuity). At the same time, according to the MRI data, tumor mass was not detected; however, the infiltration and thickening of the meninges of the brain were noted, as well as the signs of occlusive triventricular hydrocephalus and periventricular cerebral edema. Importantly, some advanced MRI protocols, such as diffusion tensor imaging (DTI), and simple quantified basic MRI sequence analysis are successfully used for the differentiation of medulloblastomas, glial tumors, and ependymomas (19, 20).

Furthermore, in the absence of pathological changes according to the examination of CSF, as well as the exclusion of bacterial, tuberculous, viral and fungal brain lesions, and autoimmune encephalitis (confirmed by the presence of antibodies in peripheral blood and cerebrospinal fluid to NMDA receptors), the patient must be referred to a specialized oncological hospital to exclude cancer. Early biopsy with subsequent histological examination should be performed to verify the diagnosis and select the treatment options.

It is necessary to underline that in our case, the lack of tumor samples was a limiting factor for using standard diagnostic technologies. Our objective was to provide an integrated diagnosis with molecular analysis considering the rarity of the form of MB.

Another important problem that should be addressed is the lack of sufficient publicly available databases containing genomic sequencing data of patients with MB, in particular with the primary leptomeningeal form of MB. A biopsy sample from the patient was used for targeted next-generation sequencing (NGS) of the previously described cancer-related genes (21). The subsequent NGS data analysis was performed using previously described tools (22–28), which revealed in total 1,808 nucleotide sequence variations (Supplementary Table 1). Nine of the identified single-nucleotide variants (SNVs), within the coding sequences of the *BRCA2*, *CHEK2*, *ERBB2*, *FLT3*, *IDH1*, *KDR*, *MC1R*, *RNF43*, and *SLC45A2* genes, were considered to be potentially significant (29). To classify the molecular subgroup of MB, the nucleotide sequence variations found in the patient’s biopsy sample were compared with those identified in a whole-genome study of 491 MB samples (30). Since such comparisons did not identify a single overlapping mutation, the affected genes were then compared. In total, 36 genes were common between the case tumor sample and the drivers identified in the whole-genome study (Supplementary Table 2). However, the comparison of the patterns of gene mutations in the molecular subgroups of MB (30) with that in the case tumor sample resulted in very low Pearson’s correlation coefficients (0.099, −0.157, 0.047, and −0.070) between the studied sample and the WNT, SHH, Group 3, and Group 4 subgroups of MB, respectively (Supplementary Table 2). Thus, the classification of the molecular subgroup for the studied sample of MB appeared to be impossible. We consider that to determine the subgroup of MB, it is necessary to expand the number of studied genes and study as many clinical samples as possible. Subsequently, this can facilitate both accurate subtyping of the tumor and the timely selection of the optimal therapy.

## Conclusion

Diagnosis of primary leptomeningeal MB is challenging due to non-specific clinical symptoms, atypical radiological picture, and rapidly progressive nature of the disease that requires early biopsy with a subsequent histological examination to verify pathological process and initiate therapy. Primary leptomeningeal MB harbor unfavorable outcome that is explained by diagnostic difficulties, rapid clinical deterioration, and unsatisfactory therapy response. Also, it cannot be ruled out that unfavorable outcome is explained by specific somatic mutations in tumor or hereditary cancer predisposition syndrome.

## Data Availability Statement

The dataset presented in this study can be found in Mendeley Data repository (doi: 10.17632/98nt6b9dvc.1).

## Author Contributions

YD and AK performed the diagnostics and treatment of the patient. YD, DM, AD, ASt, and APe contributed to the conceptualization of the study. DM, YD, ASm, AD, DR, LM, ASt, OF, OS, and APe wrote and edited the manuscript. EO and APi performed the bioinformatic analysis of the NGS data. All authors participated in group discussions and in commenting upon drafts of the manuscript.

## Conflict of Interest

The authors declare that the research was conducted in the absence of any commercial or financial relationships that could be construed as a potential conflict of interest.

## Publisher’s Note

All claims expressed in this article are solely those of the authors and do not necessarily represent those of their affiliated organizations, or those of the publisher, the editors and the reviewers. Any product that may be evaluated in this article, or claim that may be made by its manufacturer, is not guaranteed or endorsed by the publisher.

## Supplementary Material

Supplementary Figure 1 Contrast-enhancing CNS MRI (CE T1-WI). MRI showing diffuse meningeal enhancement (green arrows) in cerebral hemispheres **(A)** and posterior cranial fossa **(B)**.

Supplementary Figure 2 CNS MRI **(A)**. T2-WI **(B)**. TIRM zones of edema, hemorrhagic impregnation, cortical cystic lesions.

Supplementary Figure 3 CNS MRI TIRM. VPS shunt in the left lateral ventricle, periventricular edema; subarachnoid space is narrowed.

Supplementary Figure 4 Spinal cord MRI (T2-WI).

Supplementary Figure 5 CNS MRI of the patient after 5.5 months of the debut of the disease. **(A)** Brain MRI (post-contrast CE T1-WI). **(B)** Brain MRI (post-contrast CE T1-iso-WI). Accumulation of contrast agent by thickened meninges in cerebellar tentorium.

Supplementary Figure 6 CNS MRI of the patient after 5.5 months of the debut of the disease. Brain MRI (T2-WI).

Supplementary Table 1 Nucleotide variations identified in the tumor sample by targeted NGS. Raw NGS data were generated using NextSeq 500 System (Illumina, United States) and subjected to the standard analysis, which included FASTQ data generation, quality filtering, and mapping to the human HG19 (GRCh37.p13) genome assembly using bowtie2 (22) (http://bowtie-bio.sourceforge.net/bowtie2/index.shtml), that resulted in the generation of BAM file for the tumor sample. MuTect2 algorithm from the GATK toolset (23) (https://gatk.broadinstitute.org/hc/en-us/articles/360037593851-Mutect2) in “tumor only” mode was used to identify mutations in the BAM file, resulting in the generation of the unfiltered output VCF file. Filtration of raw output of Mutect2 was made using the FilterMutectCall tool from the GATK toolset (23) (https://gatk.broadinstitute.org/hc/en-us/articles/360036856831-FilterMutectCalls) with -- min-allele-fraction 0.4. To remove all mutations that did not pass the filter and with read depth less than 15 (-- min-meanDP 14), we used VCFtools (24) (https://vcftools.github.io/index.html). Annotation of identified mutations was done with the SnpEff tool (25) (http://pcingola.github.io/SnpEff/). The potential pathogenic significance of nucleotide variations was evaluated using the CADD tool (https://cadd.gs.washington.edu/). Particularly, mutations were ranged according to scaled PHRED-like CADD score (PHRED) (26, 29), PolyPhen category of change (PolyPhenCat) (27), and the Sorting Intolerant From Tolerant algorithm (SIFTcat) (28).

Supplementary Table 2 Attempt to classify the molecular subgroup of the case tumor sample based on the identified nucleotide variations. To properly compare the patterns of gene mutations in the case tumor sample and in the driver genes defined for molecular subgroups of MB earlier (30), we first processed single-nucleotide variants (SNVs) and insertions/deletions (InDels) identified (30) exactly in the same way as it was done for nucleotide variations found in the case tumor sample. Next, genes with at least one mutation with CADD PHRED-like value (PHRED) < 15 were arbitrarily assigned a score of 0.5; genes with at least one mutation with PHRED ≥ 15 and classified by PolyPhenCat as “probably_ damaging” or “possibly_damaging” were arbitrarily assigned a score of 2.0; other genes with at least one mutation with PHRED ≥ 15 were arbitrarily assigned a score of 1.0. Then, using the information on the frequency of mutations in each gene in molecular subgroups of MB, we computed the weighted score of each driver gene in each molecular subgroup, by multiplying the arbitrarily assigned scores by mutation frequencies. A comparison of gene scores obtained for the studied tumor sample with the weighted scores computed for the driver genes was performed by calculating the Pearson’s correlation coefficients.

## Abbreviations

MB: medulloblastoma
CNS: central nervous system
MRI: magnetic resonance imaging
VPS: ventriculoperitoneal shunt.
