# Supplementary material for: Case Report: Primary Leptomeningeal Medulloblastoma in a Child: Clinical Case Report and Literature Review
Source: Front Pediatr. 2022 Jul 11;10:925340. doi: 10.3389/fped.2022.925340 (PMC9309486; doi:10.3389/fped.2022.925340)
Supplement: Supplementary Figure 1 — Contrast-enhancing CNS MRI (CE T1-WI). MRI showing diffuse meningeal enhancement (green arrows) in cerebral hemispheres (A) and posterior cranial fossa (B). [file Data_Sheet_1.pdf]

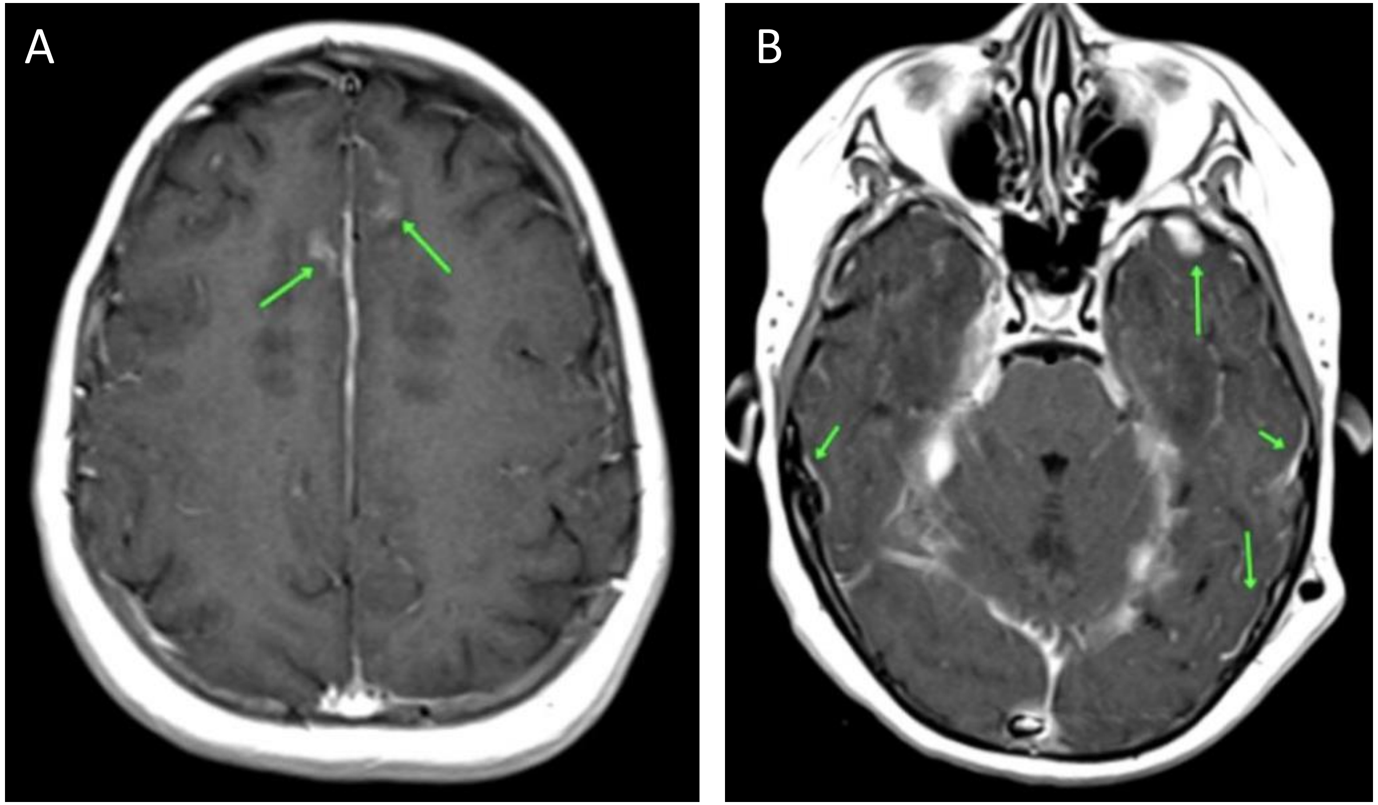

Supplementary Figure S1

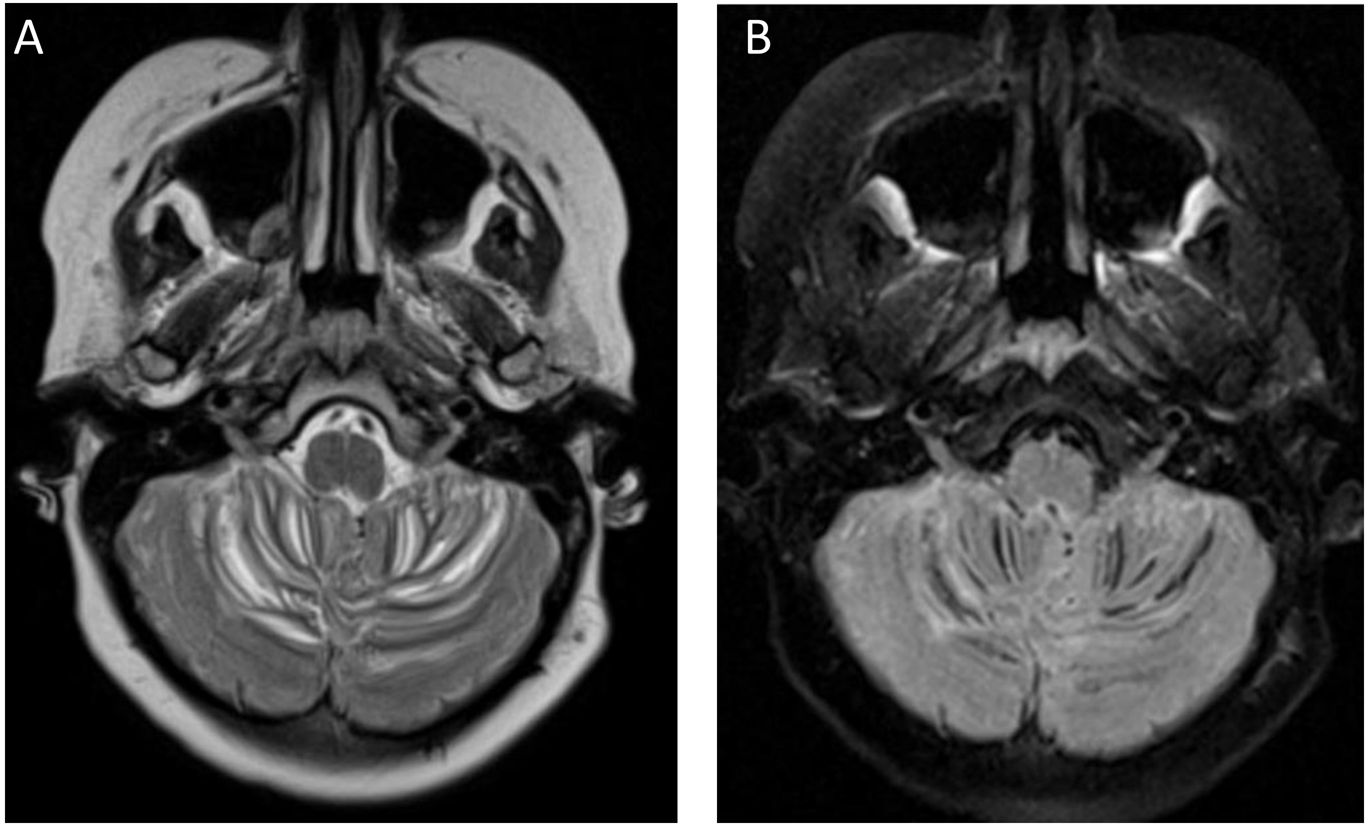

Supplementary Figure S2

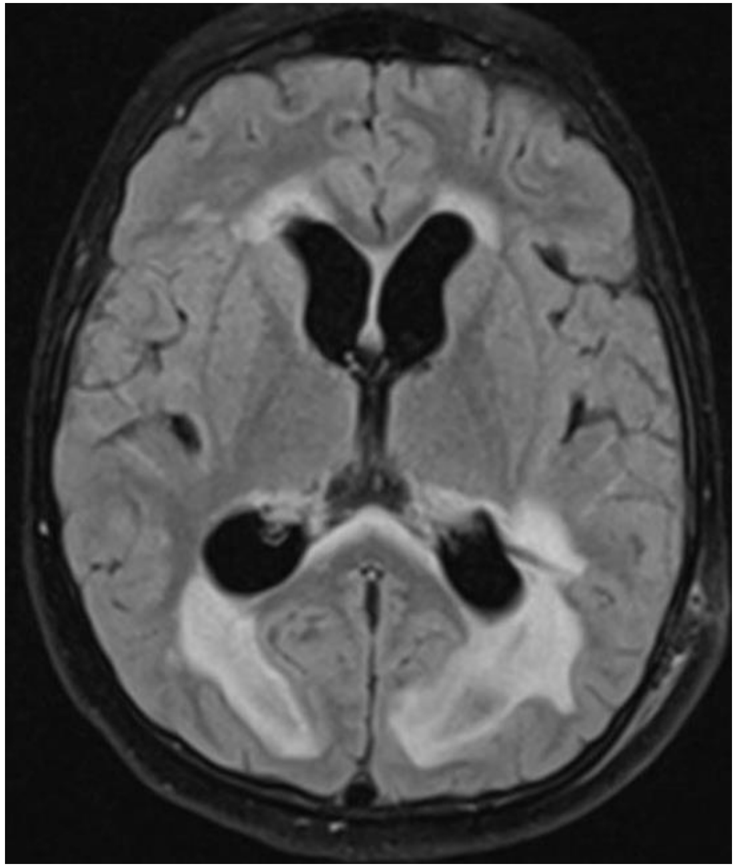

Supplementary Figure S3

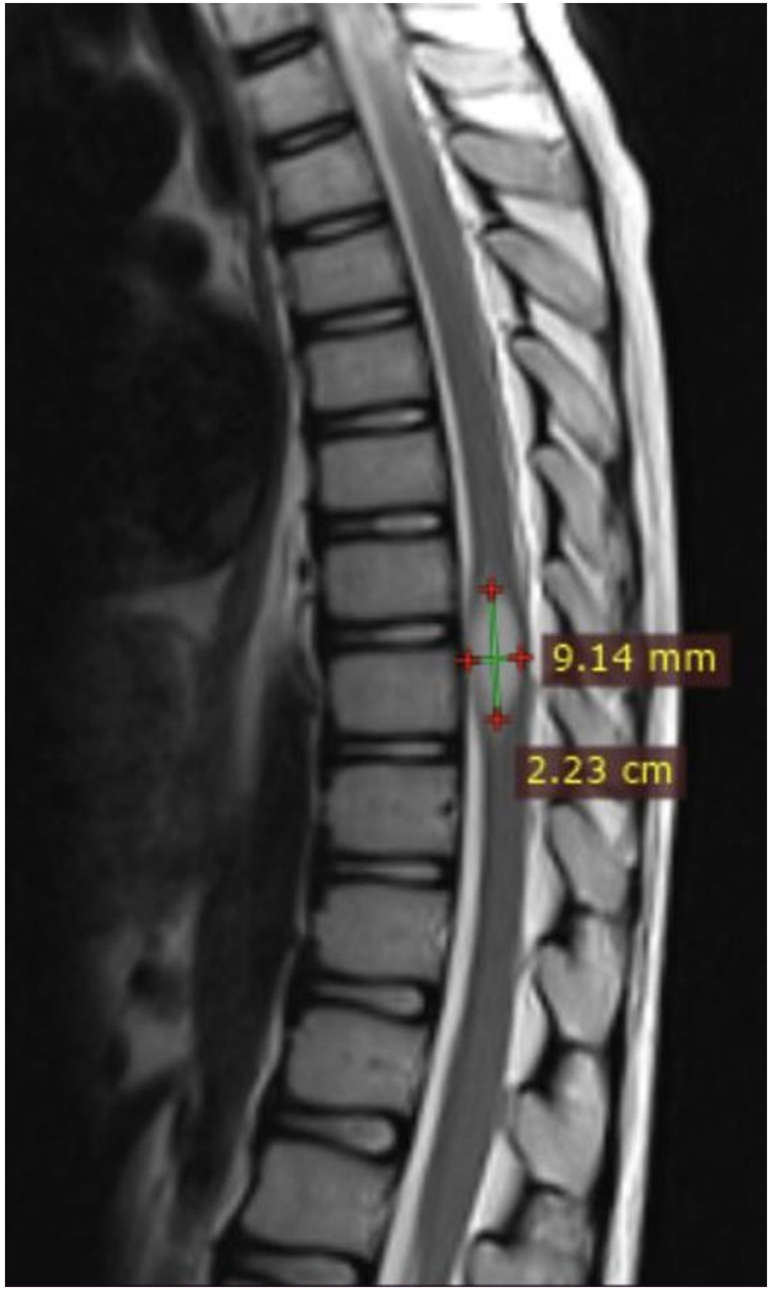

Supplementary Figure S4

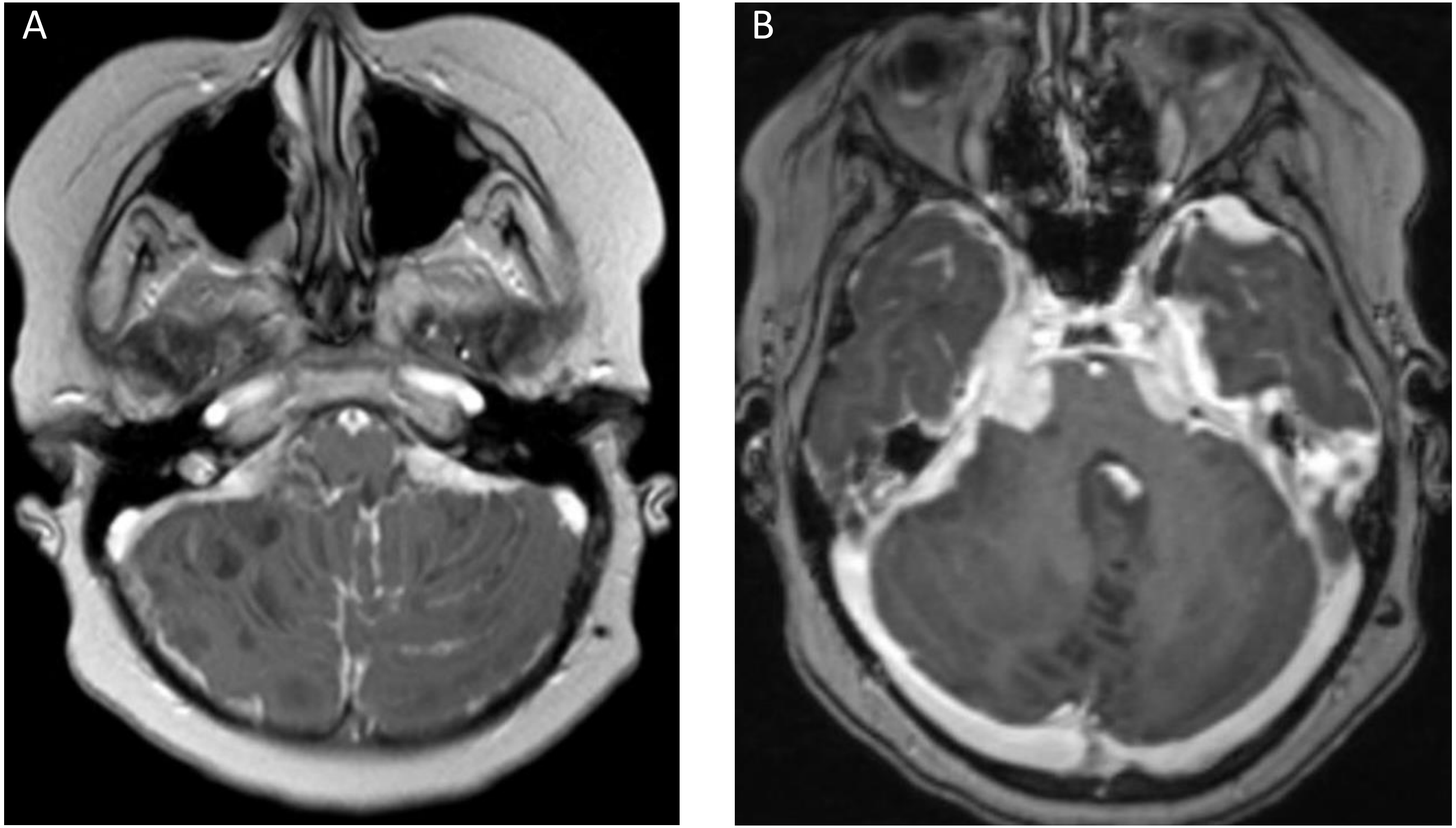

Supplementary Figure S5

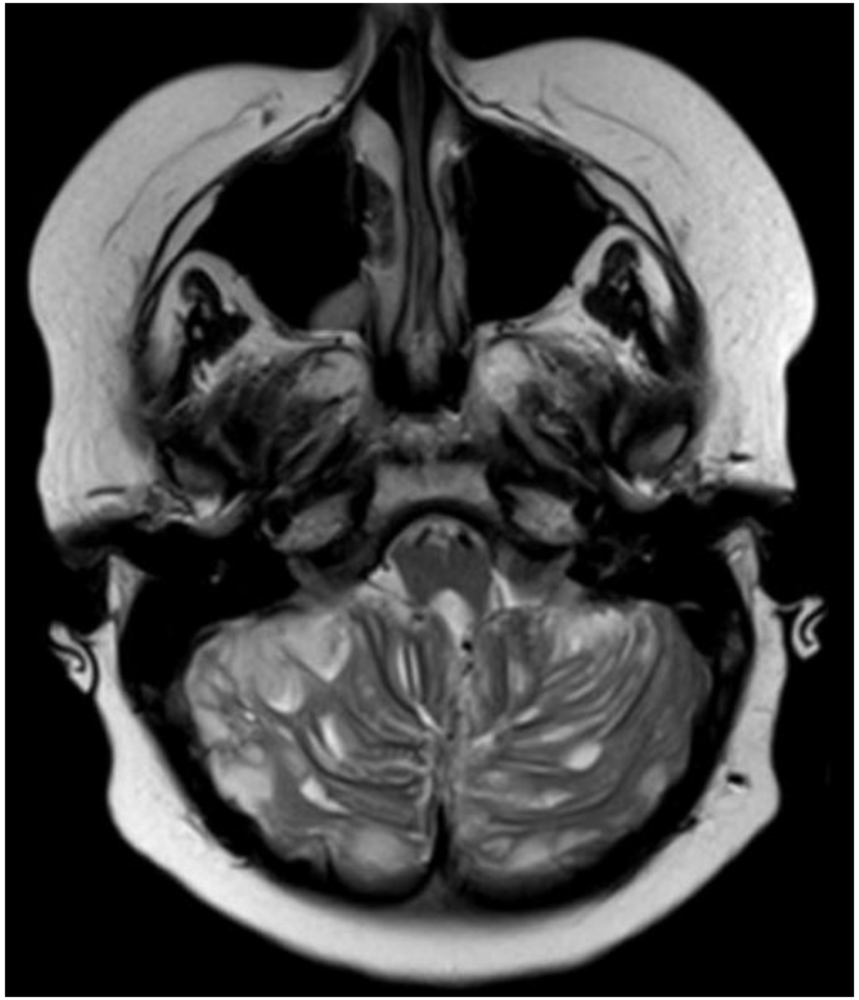

Supplementary Figure S6
